# Risk Factors for End-Stage Renal Failure Among Patients on Hemodialysis in Aljomhory Hospital, Sa’adah Governorate, Yemen: Hospital-Based Case-Control Study

**DOI:** 10.2196/14215

**Published:** 2019-09-25

**Authors:** Mohammed Dahnan, Ali M Assabri, Yousef S Khader

**Affiliations:** 1 Yemen Field Epidemiology Training Program Sa'adah Yemen; 2 Faculty of Medicine and Health Sciences Sana'a Yemen; 3 Faculty of Medicine Jordan University of Science & Technology Irbid Jordan

**Keywords:** renal failure, end-stage renal failure, risk factors, case-control study, Yemen

## Abstract

**Background:**

More than 16% of the world’s population is affected by chronic kidney disease, and these people are at the highest risk of developing end-stage renal failure (ESRF).

**Objective:**

The aim of this study was to determine the risk factors of ESRF in Sa’adah Governorate in Yemen.

**Methods:**

A hospital-based case-control study (86 cases and 263 controls) was conducted in the Aljomhory Hemodialysis Center in Sa’adah city, Yemen. Patients with ESRF who attended the hemodialysis center in Aljomhory Hospital in Sa’adah City from January 1 to February 15, 2016, were included. Control participants were healthy persons without end-stage renal disease (ESRD) who attended Aljomhory Hospital as outpatients’ relatives during the study period.

**Results:**

A total of 86 cases and 263 controls were included in this study. The mean age was 43.3 (SD 17.7) years for cases and 32.3 (SD 13.0) years for controls. In univariate analysis of factors associated with ESRD, patients aged≥40 years were 3.7 times more likely to have ESRD than younger patients. The odds of ESRD was higher among men than women. Illiteracy was significantly associated with higher odds of ESRD. Hypertension (odds ratio [OR]=8.34), diabetes (OR=3.07), cardiovascular diseases (OR=12.71), presence of urinary stones (OR=21.87), recurrent urinary tract infection (OR=9.64), cigarette smoking (OR=2.44), and shammah use (OR=6.65) were significantly associated with higher odds of ESRD. Hypertension (OR=6.68), urinary stones (OR=16.08), and recurrent urinary tract infection (OR=8.75) remained significantly associated with ERD in multivariate analysis.

**Conclusions:**

Hypertension, presence of urinary stones, and recurrent urinary tract infections were significantly associated with ESRF development. Improving the management of hypertension and designing suitable interventions to control problems of the urinary tract would help reduce ESRD prevalence.

## Introduction

Chronic renal failure (CRF) or end-stage renal failure (ESRF) is defined as a permanent reduction in the glomerular filtration rate (GFR), sufficient to produce detectable alterations in the patient’s wellbeing and organ function [1]. ESRF is defined as a GFR<15 mL/min/1.73 m² or very high albuminuria (>300 mg albumin/24 h) [2,3]. Up to 16% of the adult population internationally are affected by chronic kidney disease (CKD) [4]. More than 1.4 million patients are receiving renal replacement therapy (RRT) globally, with the annual incidence rate reaching 8% [3]. Kidney disease is the ninth leading cause of death in the United States [5]. High end-stage renal disease (ESRD) prevalence rates have been reported in many countries worldwide [6-8].

In 2006, the average incidence rate of ESRD in 10 countries in the Eastern Mediterranean Region, including Yemen, was 93 patients per million people [9]. The lowest prevalence was in Kuwait, with 80 patients per million people, and the highest was in Saudi Arabia and Yemen, with 462 and 320 patients per million people, respectively. Diabetes mellitus was the most frequently reported cause of ESRD in almost all countries, accounting for 20%–40% of the cases, followed by hypertension (accounting for 11%–30%) and glomerulonephritis (accounting for 11%–24%) [9].

CRF is a growing problem in Yemen. Between January 1998 and December 2002, 547 patients were admitted to the Science & Technology University Hospital, Sana’a (the capital city) including children with acute renal failure and CRF. CRF was observed in 400 patients, with an incidence of 64 per million people per year and a prevalence of 320 per million people. Acute renal failure occurred in 147 persons, with an incidence of 23.5 per million per year and a prevalence of 117.5 patients per million people. Of all patients, 72% were adults (age range, 20-60 years) with a male preponderance. As Yemen is a tropical country, malaria (27.9%), diarrhea (13.6%), and other infectious diseases were the main causes of renal failure [10]. The incidence might probably be higher in other governorates in Yemen, such as Hodeidah, because of the high prevalence of malaria, schistosomiasis, and renal stones and a low socioeconomic status [10]. In Yemen, the mortality was high in patients with malaria and those with associated hepatocellular failure [11]. The aim of this study was to determine the risk factors of ESRF in Sa’adah Governorate in Yemen.

## Methods

This was a hospital-based case-control study. Cases include both previously and newly diagnosed ESRD patients who attended the hemodialysis center in Aljomhory Hospital in Sa’adah City during the study period, from January 1 to February 15, 2016. Control participants were healthy persons without ESRD who attended Aljomhory Hospital as outpatients’ relatives during the study period. All cases were included, and systematic random selection of controls (a third person was involved to enable the researcher to complete interviews of each person) was performed. The sample size was calculated using Open Epi (version 3.0, Centers for Disease Control and Prevention, Atlanta, Georgia) with 95% CI and 80% power. Using a case-to-control ratio of approximately 1:3, the sample size to detect an association with an odds ratio (OR) of 2 between any exposure and ESRD was estimated as 349 participants.

ESRF was defined according to the American National Kidney Foundation Definition (GFR<15 mL/min/1.73 m²) or very high albuminuria (>300 mg/24 h) or as a serum creatinine level>3 mg/dL [12]. Data were collected using face-to-face interviews and a structured questionnaire. The questionnaire included questions about demographic characteristics, medical history, and family history.

Ethical clearance was obtained from the Ethics Committee in the Ministry of Public Health and Population prior to data collection. Participation in the study was voluntary. Data were entered and analyzed using Epi Info (Centers for Disease Control and Prevention, Atlanta, Georgia) A Chi-square test was used to compare the percentages, and an independent *t* test was used to compare means. Binary logistic regression was used to determine the factors associated with ESRF. A *P* value<.05 was considered statistically significant.

## Results

A total of 86 cases and 263 controls were included in this study. The mean age was 43.3 (SD 17.7) years for cases and 32.3 (SD 13.0) years for controls. The highest proportion of ESRF in our study was observed in the age group of ≥60 years, constituting 29.1% of the cases. Table 1 shows the sociodemographic, clinical, and relevant characteristics of patients and controls. Almost half of the cases (n=46, 53.5%) and controls (n=62, 23.6%) were aged≥40 years. About 39 (45.3%) cases and 161 (61.2%) controls were female. The proportion of hypertension was significantly higher among cases than among controls (48.8% vs 10.3%, *P*<.001). The cases were significantly more likely to have urinary stones (40.7% vs 3.0%, *P*<.001) and recurrent urinary tract infection (79.1% vs 28.1%, *P*<.001) than the controls.

**Table 1:** The sociodemographic, clinical, and relevant characteristics of patients and controls.

Characteristic		Cases, n (%)	Control, n (%)	*P* value
**Age (year)**				<.001
	<40	40 (46.5)	201 (76.4)	
	≥40	46 (53.5)	62 (23.6)	
**Education**				.005
	Illiterate	57 (66.28)	126 (47.91)	
	Literate	29 (33.72)	137 (52.09)	
**Gender**				.01
	Male	47 (54.7)	102 (38.8)	
	Female	39 (45.3)	161 (61.2)	
**Presence of hypertension**				<.001
	Yes	42 (48.8)	27 (10.3)	
	No	44 (51.2)	236 (89.7)	
**Presence of diabetes mellitus**				.011
	Yes	11 (12.8)	12 (4.6)	
	No	75 (87.2)	251 (95.4)	
**Presence of cardiovascular diseases**				<.001
	Yes	11 (12.8)	3 (1.1)	
	No	75 (87.2)	260 (98.9)	
Presence of urinary stones				<.001
	Yes	35 (40.7)	8 (3.0)	
	No	51 (59.3)	255 (97.0)	
**Recurrent urinary tract infection**				<.001
	Yes	68 (79.1)	74 (28.1)	
	No	18 (20.9)	189 (71.9)	
**Cigarette smoking**				.005
	Yes	24 (27.9)	36 (13.7)	
	No	62 (72.1)	227 (86.3)	
**Shammah use**				<.001
	Yes	14 (16.3)	8 (3.0)	
	No	72 (83.7)	255 (97.0)	

Table 2 shows the univariate and multivariate analyses of factors associated with ESRD.

In the univariate analysis of factors associated with ESRD, cases were significantly more likely to be illiterate than controls, and patients aged ≥40 years were 3.7 times more likely to have ESRD than younger patients. Patients of male gender had significantly higher odds of developing ESRD compared to those of female gender. Illiteracy was significantly associated with ESRD. Hypertension (OR=14.13), diabetes (OR=3.07), cardiovascular disease (OR=10.24), presence of urinary stones (OR=24.76), recurrent urinary tract infection (OR=14.13), cigarette smoking (OR=7.39), and shammah use (OR=6.65) were significantly associated with higher odds of developing ESRD.

In multivariate analysis, hypertension (OR=6.7), presence of urinary stones (OR=16.1), and recurrent urinary tract infection (OR=8.7) were the only factors associated with ESRD after adjusting for other variables.

**Table 2:** Univariate and multivariate analysis of factors associated with end-stage renal disease.

Factor	Univariate analysis		Multivariate analysis	
	OR^a^ (95% CI)	*P* value	OR (95% CI)	*P* value
Age (≥40 vs <40 years)	2.24 (3.73-6.21)	<.001	2.2 (0.91-5.34)	.08
Education (illiterate vs literate)	1.58 (0.97-2.6)	<.001	1.1 (0.45-250)	.87
Gender (male vs female)	1.9 (1.16-3.1)	.01	1.7 (0.75-3.87)	.20
Hypertension (yes vs no)	8.34 (4.67-14.91)	<.001	6.7 (2.7-16.4)	<.001
Diabetes mellitus (yes vs no)	3.07 (1.03-7.23)	.012	0.56 (0.14-2.21)	.39
Cardiovascular disease (yes vs no)	12.71 (3.45-47)	<.001	3.6 (0.8-17.3)	.10
Urinary stones (yes vs no)	21.87 (9.58-50)	<.001	16.1 (5.7-45.4)	<.001
Recurrent urinary tract infection (yes vs no)	9.64 (5.37-17.13)	<.001	8.7 (4.2-18.3)	<.001
Cigarette smoking (yes vs no)	2.44 (1.35-4.39)	.005	1.0 (0.39-2.65)	.96
Shammah use (yes vs no)	6.19 (2.5-15.34)	<.001	2.2 (0.50-9.33)	.30

^a^OR: odds ratio.

## Discussion

The highest proportion of ESRF in our study was observed in the age group of >60 years, which is similar to the data reported in the United States Renal Data System 2012 Annual Data Report Atlas of ESRD, which showed a predominance of ESRF among people above the age of 60 years [6]. In addition, our findings are in agreement with those of other case-control studies in which the mean age for patients with ESRF was 64 years. Other studies have also reported similar mean ages for patients with ESRF [3,12].

There was a slightly higher percentage of men among the cases in our study (55%). A study in Ivory Coast showed that male patients with ESRD constituted 61% of the study population [1]. In a case-control study conducted in Saudi Arabia and Egypt, male ESRD patients constituted 65% [13] and 61% [14] of the study population, respectively.

Illiteracy was associated with an increased odds of ESRF in our study. This finding is similar to that reported in a case-control study conducted in Taiwan, which reported a strong association between illiteracy and ESRF (OR=2.78, 95% CI 1.49-5.19) [15].

Hypertension and diabetes mellitus were associated with ESRF in our study. A case-control study conducted in Taiwan showed a similar significant association between ESRF and hypertension (OR=4.23, 95% CI 2.51-7.13) as well as ESRF and diabetes mellitus (OR=7.45, 95% CI 3.54-15.53) [15]. Another case-control study conducted in Gujrat, Pakistan, found that hypertension is associated with ESRF (OR=15.16, 95% CI 7.116-32.324) and diabetes mellitus (OR 11.2, 95% CI 5.337-23.620) [16]. The same finding was reported in Arar City, Saudi Arabia, wherein hypertension (OR=6.17) and diabetes mellitus (OR=2.14) were associated with ESRF [17].

In agreement with other studies’ findings [17,18], our findings showed that there is significant association between cardiovascular diseases and ESRF. We also noted that kidney or urinary tract stones were potentially associated with ESRF development. This finding was reported in other studies as well [19,20]. Regarding the strong association of recurrent kidney or urinary tract infection and ESRF, our findings are in agreement with those of other studies conducted in Pakistan [16] and Saudi Arabia [15].

Unlike many studies [21-24], this study showed no association between frequent analgesic intake and ESRF. Regarding the use of tobacco, our findings are similar to the findings of several studies that indicate that tobacco use (cigarette smoking) is considered a risk factor for ESRF [14,19,25,26].

In conclusion, hypertension, recurrent urinary tract infection, urolithiasis, family history of ESRF, and diabetes mellitus were potential risk factors for ESRD in the Yemeni community.

## Abbreviations

CKD: chronic kidney disease
CRF: chronic renal failure
ESRF: end-stage renal failure
ESRD: end-stage renal disease
GFR: glomerular filtration rate
OR: odds ratio
